# International cooperation to promote advances in medicine

**DOI:** 10.4103/1817-1737.41913

**Published:** 2008

**Authors:** Ghazwan Butrous

**Affiliations:** *University of Kent, Canterbury, Kent, United Kingdom*

Medical and scientific researches in the last few years have become increasingly global, cross-national, cross-cultural, and collaborative. Today, one fifth of the medical and scientific papers have international coauthors. This reality is a reflection of globalization of modern day life and easy communications and movement of health professionals and patients. International collaboration is emerging as a norm and is an accepted form of current research environment. It is strongly encouraged by funding agencies.

The sheer volume of international collaborations has been increased substantially during the last 20 years.[1,2] This increase is also noticed in the number of internationally co-authored papers between the developed (usually referred to in political jargon as the ‘North’) and developing and emerging countries (usually referred to as the ‘South’, for example, India and Bangladesh, Mexico and Brazil, China and Pakistan). Despite its late start, China has published many collaborative papers with most Asian countries and also with the North, confirming the effectiveness of China's current open-door policy. Papers resulting from international collaboration appear in higher-impact journals and are cited more often than papers that are the outcome of local research.[3] Chen *et al.*[2] reported that only 7.4% of the papers appearing in journals with an impact factor equal to or greater than 5 were published by the local authors during the study period in Taiwan (1990-2004) compared to 13.6% of the publications that had international collaboration. Taiwan's researchers collaborated with 76 countries, with the greatest share from USA (69.9%). Although the majority of collaborations are mainly between the North and the South, only one fifth are South-South collaborations (collaboration between developing countries).[4]

However, majority of these collaborations are still disorganized efforts, and they vary according to the centers and universities they are originating from. There are no coherent international collaborations, and many failures have been reported. We can summarize the patterns of collaboration as follows:

1. Short-term project-oriented collaboration, where a few centers in different countries are working together for one project. This is often controlled by the availability and the policy of funding agencies.
2. Long-term collaboration, forming an ‘alliance’ between research organizations who share the same interest. There are no rules to govern these activities, which are carried out on an ad hoc basis. It is most likely to happen amongst people who have trained in the same center or are sharing the same interest.
3. Organized international collaboration, which is usually initiated by centers of excellence trying to find new research (and may be commercial) opportunities in a promising emerging market. We are seeing an increase in this form of collaboration, where centers of excellence in the developed countries are opening research offices to facilitate and enhance continuous collaborations. These centers are targeting opportunities in many developing countries such as the Middle East, China, and Southeast Asia.
4. The large, learned scientific societies are now an accepted platform of international collaborations. American Thoracic Society is an example. It attracts delegates from all over the world, and it has become more international than ‘American.’ The 2007 American Thoracic Society conference in San Francisco was attended by over 16,000 people from 90 countries. Unfortunately, most of the sponsorship and attendance is governed by commercial interest of the pharmaceutical companies rather than scientific motivation and need. There is also a trend emerging whereby these regional societies are forging a very close scientific collaboration with each other, for example, the close association of American Thoracic Society and European Respiratory Society, which results in many combined guidelines and expert documents. Many such collaborations are present in the genetic field of infectious diseases,[5] but most are in infant stages and need further support and funding.
5. International cross-border activities such as the Global Alliance Against Chronic Respiratory Diseases (GARD) http://www.who.int/respiratory/gard/en/; and *Global Strategy for Asthma Management and Prevention* http://www.ginasthma.com/.
6. A growing interest towards conducting clinical trials and involving developing countries, where commercial organizations, in particular, pharmaceutical companies, are becoming increasingly active in this field.
7. There is an increasing trend of establishing an equal partnership with scientists from both the North and the South. For example, we have noticed the need to collaborate in ‘pulmonary vascular disease’ on a global scale. This condition started to attract attention, and more patients were diagnosed as therapies started to be available. Although the condition may affect less than 150,000 people in the west, it is far more common in the developing world (with an estimate of more than 25 million people) because of the other etiological factors which are not seen in the western environment. Unfortunately, the commercial power in marketing drugs has helped in shaping the condition to the extent that it only reflects the interest of a few in the North and hinders progress of the many. This situation prompted the formation of a virtual institute for pulmonary vascular diseases, (www.pvri.info), which includes equal partnership of experts and researchers from the North and the South.
8. Collaboration as a result of the growing global health threats, including climate changes, AIDS, tuberculosis, malaria, chronic obstructive lung diseases, and the easy spread of these conditions, as we have seen recently with avian flu. This makes the collaboration between nations for research and exchange of knowledge and information not only a luxury but a necessity.

International Research Corporation has always helped scientists to keep abreast of international science and share expertise and resources[6] which enhance the scientific community and in-house training. It benefits both the health care system and the population as it may provide new treatments which are probably not already available in that country. It also helps in building up of research capacity and has direct economic significance. Some governments are already beginning to pay premiums to become hubs in the global excellence network. It remains to be seen whether this development will produce significant changes in the world research capacity.

The benefits have also been reflected in the developed countries.[4,7,8] For example, the new policies in the United Kingdom which are led by primary health care have resulted from international health policy based on the experiences of many developing countries over the past 30 years.[8]

The increasing collaborations on human subject research have crossed international borders and created a number of issues and raised various questions, particularly in the developing world. These include ethical, cultural and ethnic issues.

The proportionally heavy burden of diseases, particularly infectious diseases, and other environmental factors may affect the results of the studies. The underlying conditions prevalent in the study area may not necessarily apply to the conditions in other countries. For example, in chronic obstructive lung disease, smoking is the most common cause of this condition in the west; the cause may not necessarily be the same in India, where it could be due to cooking fumes, for example; or due to sandstorms in countries such as the Middle East or Sub-Saharan Africa. Furthermore, the presence of a core infection may change the pathophysiological milieu of the disease, which may result in a different response to the treatment or to the immunological or the pathophysiological reaction, which makes it difficult to translate the results to the western developed world.

The ethical issue is the most important issue involved in cooperation with the developing world. This is a very complex subject, and it was reviewed by Varmus and Satche and others.[4,9-11] Although many studies claimed that they adhere to the international guidelines, it is important to consider the details of these guidelines, the socioeconomic and cultural structure of the country. In response to this reality, international organizations started task forces to guide doctors and investigators in the complex world of human subject research. Guidelines were produced, including the declaration of Helsinki; International Ethical Guidelines for Biomedical Research Involving Human Subjects;[12] the guidelines of Good Clinical Practice; and the guidelines from the International Conference of Harmonization http://www.ich.org/. There are also specific guidelines regarding certain diseases or conditions, such as epidemiological studies, genetics, biomedical and pharmaceutical trials. Clinical practice guidelines are regarded as powerful tools to achieve effective health care. Although many countries have built up experience in the development, appraisal, and implementation of guidelines, there has been no established forum for collaboration at an international level. In an effort to partly resolve this issue, in 2002 the international Guidelines International Network (GIN) was formed http://www.g-i-n.net/. This network aimed at promotion of systematic guideline development and implementation which ‘*seeks to improve the quality of health care by promoting systematic development of clinical practice guidelines.*’ There are also other similar organizations aiming to develop, teach, and promote evidence-based health care and provide support and resources to anyone who wants to make use of these, such as Oxford Centre for Evidence-based Medicine http://www.cebm.net/.

Funding is a big issue which needs to be considered seriously considering that more than 90% of the world's ‘potential years of life lost (PYLL)’ belong to the developing world, but it attacks less than 10% of global research funds.[13,14] Indeed, current funding, averaging around US$ 20,000, is too low even to ensure that a single country's research is successful.[15] Although there are increasing funding opportunities, mainly via philanthropic organizations, some NGOs, and even governmental organizations, the sheer problem is so huge that more is continually needed.

While minimum research capacity may exist in many developing countries, the fact that lead institutions, as well as study countries, are concentrated in a handful of centers attests to great disparities in research capacity. Mechanisms must be introduced to ensure investment in a research capacity, particularly infrastructure research capacity such as research nurses and research associates.[16]

Careful consideration of the cultural, social, and intellectual properties of the developing countries has played a part in maintaining a successful research partnership. Patenting of biological materials, including plants, animals, and even discoveries, made in the developing countries, whether due to existing knowledge of the local and indigenous people or by considerable input from local researchers, is becoming a key issue of contention between multinational companies and health care campaigners and other campaigners (usually referred to by the negative term biopiracy).[17] As a general rule, the sponsoring agency should agree in advance of the research that any product developed through such research will be made reasonably available to the inhabitants of the host community or country at the completion of the research. The potential for clinical research to exploit populations has raised much concern recently. It is advisable that collaborators must enter into partnership, which means that researchers must engage the population in development and evaluation of, and benefeting from the researches.[18,19] Lastly, the developing country must be empowered to participate in debate and decision making about priority setting for research collaboration. A good starting point for this is for countries in a specific region to start to collaborate to tackle common regional health problems.[19]

In conclusion, research collaboration is an enormous and powerful tool which provides mutual benefits to all parties. It needs careful planning and considerable thought to the diversity of the human society. The North-South collaboration can be strengthened by promoting further involvement of countries such as Germany, France, Belgium, and Spain in funding, supporting, and leading research in developing countries.
